# Impact of Economic Freedom and Educational Attainment on Life Expectancy: Evidence From the New EU Member States

**DOI:** 10.3389/fpubh.2022.907138

**Published:** 2022-06-30

**Authors:** Adrian Teodor Moga Rogoz, Gamze Sart, Yilmaz Bayar, Marius Dan Gavriletea

**Affiliations:** ^1^Department of Physiology, University of Medicine and Pharmacy “Iuliu Hatieganu”, Cluj-Napoca, Romania; ^2^Department of Educational Sciences, Hasan Ali Yucel Faculty of Education, Istanbul University-Cerrahpaşa, Istanbul, Turkey; ^3^Department of Public Finance, Faculty of Economics and Administrative Sciences, Bandirma Onyedi Eylul University, Bandirma, Turkey; ^4^Department of Business, Business Faculty, Babes-Bolyai University, Cluj-Napoca, Romania

**Keywords:** life expectancy, economic freedom, educational attainment, panel causality analysis, panel cointegration analysis

## Abstract

Life expectancy is a significant indicator of public health, life quality, welfare and economic development. Therefore, improvement in life expectancy is among the priority targets of the countries. This paper investigates the effect of economic freedom and educational attainment on life expectancy in the new EU member states, experiencing an institutional, educational, and economic transformation, during the period 2000–2019 by using cointegration and causality tests, because economic freedom and educational attainment can foster the life expectancy through institutional and economic variables such as institutions, governance, sound monetary and fiscal policies, economic growth, innovation, technological development, better living standards and access to superior healthcare services. The causality and cointegration analyses reveal that economic freedom and educational attainment are significant factors underlying life expectancy in the short and long term. However, educational attainment is found to be more effective on life expectancy than economic freedom. The findings have important implications for educational and health policies in analyzed countries. Governments must understand the education–health relationship to be able to develop and promote educational policies that have the potential to improve public health.

## Introduction

Life expectancy is a significant indicator of health status and, implicitly, of the human capital stock of a country. Therefore, any increase in life expectancy reflects improvements not only in the health level of a society, but also in its human development and wellbeing. The globalized world has experienced significant improvements in life expectancy thanks to advances in health care, medical care and living standards (1). The life expectancy at birth has increased up to 73.4 years in 2019 from 66.8 years in 2000 and the healthy life expectancy at birth has also increased to 63.7 in 2019, from 58.3 in 2000 (2). However, life expectancy significantly varies among countries. While the top three countries with the highest life expectancy at birth for both sexes in 2019 were Japan (84.26 years), Switzerland (83.45 years) and, respectively, the Republic of Korea (83.3 years), the last three countries with the lowest life expectancy at birth for both sexes were Lesotho (50.75 years), Central African Republic (53.1 years) and Somalia (56.47 years) (2).

The European Union (EU) members states also experienced the improvements in life expectancy during the 2000–2019 period. Spain had the largest life expectancy at birth with 83.22 years in 2019 and Bulgaria had the lowest life expectancy at birth with 75.07 years in 2019 and the old EU members generally had the higher life expectancy at birth as seen in Table 1. However, the evolution of life expectancy between 2000 and 2019 presented in Table 1 also indicated that the new EU member states such as Estonia, Slovenia, Latvia, Hungary, Slovakia, and Poland had a relatively higher improvements in life expectancy.

**Table 1:** Evolution of life expectancy at birth in the EU states between 2000 and 2019.

**Country**	**2000 (years)**	**2019 (years)**	**Change of life expectancy (years)** **(life expectancy in 2019—life expectancy in 2000)**
Austria	78.17	81.65	3.48
Belgium	77.66	81.42	3.76
Bulgaria	71.61	75.07	3.46
Croatia	74.36	78.64	4.28
Cyprus	78.75	83.14	4.39
Czechia	74.95	79.13	4.18
Denmark	76.93	81.32	4.39
Estonia	70.94	78.88	7.94
Finland	77.6	81.61	4.01
France	78.91	82.48	3.57
Germany	78.09	81.72	3.63
Greece	78.17	81.1	2.93
Hungary	71.34	76.44	5.1
Ireland	76.41	81.84	5.43
Italy	79.36	82.97	3.61
Latvia	70.18	75.38	5.2
Lithuania	72.04	75.99	3.95
Luxembourg	78.24	82.41	4.17
Malta	77.87	81.89	4.02
Netherlands	78.01	81.79	3.78
Poland	73.7	78.27	4.57
Portugal	76.58	81.57	4.99
Romania	71.39	75.57	4.18
Slovakia	73.3	78.23	4.93
Slovenia	76.05	81.31	5.26
Spain	79.08	83.22	4.14
Sweden	79.57	82.4	2.83

*Source: World Health Organization (2)*.

The considerable differences in life expectancy have raised the attention of researchers, which started to investigate its determinants. Their results suggest that some social, economic and environmental factors, such as income level, economic growth, education, health care expenditures, number of doctors, nutrition, food availability, urbanization, environmental quality, clean water, sanitation, fertility rate, pharmaceutical consumption or tobacco consumption, could be influence factors of life expectancy (3–9).

In this study, the effect of economic freedom and educational attainment on life expectancy is analyzed. The countries with higher economic freedom have relatively larger life expectancy than the states with less economic freedom (10, 11). Economic freedom can positively influence economic growth and development, by fostering institutions, governance, legal structure, sound monetary and fiscal policies, financial development, trade liberalization, innovation and competitiveness (12–15). Meanwhile, all of these enhance life expectancy through improvements in healthcare, medical care, better access to nourishment and clean water, and environmental quality (16–18). However, when the economic growth achieved through economic freedom is accompanied by weak environmental regulations, life expectancy is negatively influenced by environmental degradation (19). Meanwhile, lower government size can negatively affect life expectancy by decreasing social expenditures (20). As a consequence, the net influence of economic freedom on life expectancy can differ depending on which factors prevail.

Educational attainment was considered a crucial factor in explaining the differences in life expectancy among countries through access to health-related information, employment of healthcare opportunities, planning horizon and risk perception at the subsequent period (21, 22). In this context, higher educational attainment generally leads to better job opportunities, with higher wages and, in turn, higher wages help individuals to have better living standards and access to superior healthcare services. Educational attainment can also foster life expectancy by raising the efficiency of health production (23). Last, educational attainment may also impact life expectancy by enhancing economic growth, many researchers suggesting that educational attainment is a significant determinant of economic growth (24, 25). Lutz and Kebede (26) investigated the relationship between education proxied by average schooling years of the adults and life expectancy and their results suggest that the education level is a better predictor of life expectancy than the other factors. Furthermore, economic freedom can influence life expectancy via educational attainment, because individuals from countries with higher economic freedom generally give more importance to educational attainment (27).

Both educational attainment and economic freedom are also significant determinants of the socioeconomic development level. Socioeconomic development levels can affect life expectancy through better nourishment and living standards and higher health care access (26). In this context, Preston (28) analyzed the relationship between life expectancy and GDP per capita and the resulting curve, named Preston curve, showed that the persons in richer countries generally had higher life expectancy than those from poor states. When the GDP per capita has low levels, the increases in GDP per capita lead to higher improvements in life expectancy. However, the improvements in life expectancy decrease at higher levels of GDP per capita. Furthermore, Preston (28) attributed the upward shifts of the curve to the advances in medical science and the health care sector.

The related empirical literature has mainly investigated the influence of GDP per capita and various educational indicators on life expectancy, but a few researchers such as Esposto and Zaleski (19, 29–32) have investigated the influence of economic freedom on life expectancy in countries with different characteristics mainly through regression approach. However, these studies have given a common effect of economic freedom on life expectancy for the whole sample and in turn an evaluation about how the influence of economic freedom on life expectancy varies among the countries cannot be made. Therefore, the limited number of studies about the interaction between economic freedom and life expectancy and their research method motivate us to analyze the influence of economic freedom together with educational attainment on life expectancy on a sample of the new EU member states, which have made significant progress in educational attainment and economic freedom thanks to the EU membership negotiations and, subsequently, adhesion by means of cointegration and causality analyses.

The scientific contribution of this article results from two aspects. First, the literature about the nexus between economic freedom and life expectancy has been quite limited. Therefore, this article will enlarge the existing literature, especially in the context in which, to the best of our knowledge, no study has investigated the influence of economic freedom on life expectancy in the case of the New EU member states. Secondly, the very limited literature has analyzed the effect of economic freedom on life expectancy by using the regression method and ignored the country-level differences. This research employed the augmented mean group (AMG) estimator to obtain the long-run effect of economic freedom on life expectancy at the country level. The rest of the article is structured as follows: the literature is summed up in Section 2, the dataset and methods are described in Section 3, empirical applications and discussions are provided in Section 4 and the conclusions are presented in Section 5.

## Literature Review

Many studies have empirically investigated the factors aimed to contribute to the improvements in life expectancy, and documented the various social, demographic, economic, environmental and institutional determinants underlying life expectancy as presented in introduction. In this study, the influence of economic freedom together with educational attainment on life expectancy is analyzed taking into account that the literature on the nexus of economic freedom-life expectance has been very limited.

The nexus between economic freedom and life expectancy has been mainly investigated by panel regression analysis in sample of countries with different economic development levels including developed, developing and underdeveloped economies. But however, the studies pointed out that economic freedom raised the life expectancy in all countries. Esposto and Zaleski (29) only checked the relationship between economic freedom and life expectancy varied based on the current life expectancy level and found that economic freedom had higher effect on life expectancy in the countries with a life expectancy under 65. The regression approach gives a common coefficient for all countries and does not let us to see how the effect of economic freedom on life expectancy varies among countries. In this study, the Augmented Mean Group estimator (AMG) was hence preferred to obtain panel and country level cointegration coefficients which indicate the long-term run effect of economic freedom on life expectancy.

In the limited empirical literature about the nexus between economic freedom and life expectancy, Esposto and Zaleski (29) investigated the effect of economic freedom on life expectancy and literacy in 92 countries, with different development levels, by using the regression method. They noticed that economic freedom increased the life expectancy and literacy. Moreover, they argued that the effect of economic freedom on life expectancy was relatively higher in the countries with a life expectancy under 65. On contrary, Gwartney and Lawson (30) suggested that people in the countries with the highest economic freedom had relatively higher life expectancy than the others.

Hassan et al. (31) investigated the relationship between economic freedom and life expectancy in 7 countries during the period 2000–2010 by using the regression method and discovered a positive relationship between economic freedom and life expectancy. Lawson et al. (32), analyzed the interaction among the economic freedom, obesity and life expectancy in 135 countries in 1995 and during the period 2000–2009 with the help of a regression approach. Their findings indicate a positive influence of economic freedom on life expectancy. Last, Sharma (19) analyzed the effect of economic freedom on health indicators in 34 sub-Saharan African economies for the period 2005–2016 via a regression approach and found a positive influence of economic freedom on life expectancy.

The first empirical studies on determinants of life expectancy have generally focused on the validity of Preston curve suggesting the relationship between GDP per capita and life expectancy. However, the influence of educational attainment, a crucial factor for the differences in life expectancy among the countries, on life expectancy has begun to be investigated as of 2000 s. The empirical studies have mainly represented the education by literacy, secondary and tertiary enrollment and education index and employed the regression analysis, cointegration analysis, and cluster analysis and have found that different education indicators have a positive influence on life expectancy in the countries with different development levels (5, 8, 26, 33–42). However, Hazan (43–45) revealed an insignificant influence of educational attainment of life expectancy for some countries.

In the empirical literature about education and life expectancy, Yavari and Mehrnoosh (33) investigated the determinants of life expectancy through regression analysis and discovered that the literacy rate is a significant factor positively influencing life expectancy. Meanwhile, Kabir (34) investigated the social and economic determinants of life expectancy in 91 developing countries through the regression analysis and revealed a significant effect of education on life expectancy in developing countries. Bayati et al. (35) also investigated the socio-economic determinants of life expectancy in 21 countries from the East Mediterranean region during the period 1995–2007 through the regression analysis and found a positive influence of education on life expectancy.

Delavari et al. (5) investigated the social and economic factors impacting life expectancy in Iran during the period 1985–2013 with the help of cointegration and regression analyses. They concluded that GDP per capita, number of doctors, literacy rate, and food availability were positive determinants of life expectancy, but fertility rate had a negative impact on life expectancy. Meanwhile, Hassan et al. (36) analyzed the determinants of life expectancy in 108 developing economies during 2006–2010 by using regression analysis. They noticed that the education index is a significant determinant of life expectancy. Ketenci and Murthy (8) also investigated the determinants of life expectancy in the United States during the period 1960–2012 by using a cointegration test with structural breaks. Educational attainment and real per capita income were the most important factors influencing life expectancy.

Lutz and Kebede (26) investigated the effect of education on life expectancy in a panel of 174 countries over the 1970–2015 period, with the help of the regression analysis, suggesting a positive effect of educational attainment on life expectancy. Moreover, Hamidi et al. (37) examined the interaction between educational attainment and life expectancy in 18 MENA countries over the 1995–2009 period and found a positive effect of educational attainment on life expectancy. Raghupathi and Raghupathi (38) investigated the interaction between education and health in 26 OECD states for the 1995–2015 period and concluded that higher education level positively affected public health and life expectancy. Paramita et al. (39) explored the determinants of life expectancy in 34 provinces of Indonesia via cluster analysis based on structural equation modeling and found a positive effect of average schooling years on life expectancy.

Hendi et al. (40) investigated the relationship between education and mortality in Finland and United States and noticed a positive effect of education level on life expectancy, faster improvements being found at higher levels of education in both countries. Case and Deaton (41) explored the role bachelor's degree (BA) on life expectancy in the United States over the 1990–2018 period and revealed that persons with BA had increased life expectancy and the gap in life expectancy between the ones with BA and the ones with no-BA consistently increased during this period. A recent study was conducted by Siegel et al. (42) regarding the social determinants of remaining life expectancy in Germany, by using data of 2015–2017 period and underlined the education level as a significant determinant of remaining life expectancy.

Some researchers such as Hazan (43–45) have noticed an insignificant effect of educational attainment of life expectancy for some countries. In this context, Hazan (43) examined the interaction between life expectancy at birth and age 5 and schooling in 61 countries during the period 1960–1990 by using correlation analysis and found a positive correlation between schooling and life expectancy at birth, but insignificant interaction between schooling and life expectancy at age 5.

Bilas et al. (44) also investigated the factors influencing the life expectancy in the EU member states via regression analysis and discovered a negative effect of education on life expectancy. Gilligan and Skrepne (45) explored the determinants of life expectancy in 21 Eastern Mediterranean countries during the period 1995–2010. The authors grouped the countries by cluster analysis and then examined the determinants of life expectancy for three clusters. Literacy proved to be a positive determinant of life expectancy only in the countries from the third cluster.

Based on the information obtained from the investigated literature, the research hypotheses of the study

are:

**Hypothesis (1):** Economic freedom has a significant impact on life expectancy.
**Hypothesis (2):** Educational attainment has a significant impact on life expectancy.

## Data and Method

The paper analyzes the influence of economic freedom and educational attainment on life expectancy in the new EU Member States during the 2000–2019 period via causality and cointegration tests. In the analyses, life expectancy (LEI) is represented by the life expectancy index, calculated by UNDP (11). The life expectancy index constitutes the health dimension of the human development index and is based on life expectancy at birth. Economic freedom is proxied by the economic freedom index, developed by Fraser Institute (10) and is calculated as a combination of government size, legal system and property rights, reasonable monetary policy, trade freedom and regulation structure [see (10) for more information about the index]. Lastly, educational attainment (EDU) is represented by the education index calculated by UNDP (11). The education index is formed by means of schooling years for individuals with 25 years or more and the expected schooling years of children (11) with school age. The data of life expectancy and education index is taken from the UNDP database and the economic freedom index is taken from the Fraser Institute database. All series are annual and their period is 2000–2019 because the economic freedom index already existed for the 2000–2019 period. The econometric tests are performed by means of Gauss 12.0, EViews 11.0, and Stata 15.0. The logarithmic forms of economic freedom, educational attainment and life expectancy (LNEF, LNEDU, and LNLEI) are utilized in the analyses to eliminate the seasonality.

The influence of economic freedom (EF) and educational attainment (EDU) on life expectancy (LEI) is analyzed using a sample of the new EU member states consisting of Bulgaria, Croatia, Czechia, Estonia, Hungary, Latvia, Lithuania, Poland, Romania, Slovakia and Slovenia by following the econometric model in equation 1.


(1) $$\begin{array}{l} LEI_{it}=f\left(EF_{it},EDU_{it}\right)\\ \;(i=1,2,\ldots ,11;t=2000,\;2001,\ldots .,2019) \end{array}$$

The descriptive characteristics of the variables are depicted in Table 2. The mean of life expectancy index, economic freedom index, and education index were 0.8489, 7.4366 and, respectively, 0.8092. Both life expectancy and education were relatively more stable in the sample, but economic freedom presented a higher variation among the countries.

**Table 2:** Descriptive statistics of the dataset.

**Characteristics**	***N***	**Observations**	**LEI**	**EF**	**EDU**
Mean	11	220	0.8489	7.4366	0.8092
Median	11	220	0.8480	7.5400	0.8180
Maximum	11	220	0.9430	8.2100	0.9100
Minimum	11	220	0.7650	5.4400	0.65400
Std.Dev.	11	220	0.0389	0.4930	0.0589
Skewness	11	220	0.1147	−1.2033	−0.5691
Kurtosis	11	220	2.4592	4.8481	2.6028

The researchers investigating the influence of economic freedom on life expectancy have mainly employed the regression method as seen in literature review and in turn an inference about the relationship between economic freedom and life expectancy at country level cannot be made. In addition to this, presence of heterogeneity and cross-sectional dependence canalize us to select econometric tests which take notice of heterogeneity and cross-sectional dependence. Therefore, Westerlund and Edgerton (46) bootstrap cointegration test, AMG estimator, and Dumitrescu and Hurlin (47) causality test are chosen to investigate the influence of economic freedom and educational attainment on life expectance in short and long term.

In the analysis section, the cross-sectional dependence and heterogeneity are firstly investigated and then, the availability of unit root in three variables is explored. At the next stage, the long-run interaction among economic freedom, educational attainment and life expectancy is investigated by using the Westerlund and Edgerton (46) bootstrap cointegration test taking the availability of heterogeneity and cross-sectional dependence, heteroscedasticity and autocorrelation and produces relatively robust consequences for small samples (46). Furthermore, it prevents the endogeneity problem through fully modified ordinary least squares. The cointegration LM (lagrange multiplier) test statistic grounded on McCoskey and Kao (48) LM test is figured out as follows (46):


(2) $$LM_{N}^{+}=\frac{1}{NT^{2}}\sum_{i=1}^{N}\sum_{t=1}^{T}{\hat{w}}_{i}^{-2}S_{it}^{2}\;$$

The traditional estimators postulate that slope coefficients are constant for all cross-sections, because these estimators enable the constant terms to become different by pooling the individual groups. Therefore, all other coefficients and error variances are constant among cross-sections (49). The second important requirement for a robust estimator is to consider the absence of cross-sectional independence. The first-generation estimators such as mean group estimator of by Pesaran and Smith (50) and pooled mean group estimator by Pesaran et al. (51) take the heterogeneity into consideration, but disregard the presence of cross-sectional dependence, a common characteristic in the highly globalized world. The second-generation AMG estimator by Eberhardt and Bond (52, 53) was utilized to predict the panel and country-level cointegration coefficients in the study due to the subsistence of heterogeneity and cross-sectional dependence. The estimator of AMG takes the availability of common factors and dynamic effects of the three series into consideration and produces efficient consequences, and can be utilized in a condition of endogeneity (52).

The cointegration coefficients are also estimated by CCE-MG (Common Correlated Effects Mean Group) estimator of Pesaran (54) and IFE (Interactive Fixed Effects) estimator of Bai (55) to check the consistency and reliability of estimations by AMG estimator. CCE-MG estimator takes the unobservable common factors into consideration by adding the cross-section averages of dependent and independent variables to the regression. On the other hand, IFE estimator takes notice of heterogeneity, cross-sectional dependency, and multifactor error structure.

Lastly, causal interaction among economic freedom, educational attainment, and life expectancy was analyzed by the causality test of Dumitrescu and Hurlin (47), an improved version of the conventional Granger causality test in a condition of heterogeneity. The causality test (x is a Granger cause y) with stationary x and y variables is defined as following (47):


(3) $$\begin{array}{r} Y_{i,t}=\alpha_{i}+\overset{k}{\underset{k=1}{\sum }}Y_{i}^{\left(k\right)}Y_{i,t-k}+\overset{k}{\underset{k=1}{\sum }}\beta_{i}^{\left(k\right)}X_{i,t-k}+e_{i,t} \end{array}$$

To sum up, Westerlund and Edgerton (46) bootstrap cointegration test is a second-generation test and takes the cross-sectional dependence unlike the first-generation cointegration tests and it also produces robust consequences under the presence of heteroscedasticity, autocorrelation, and endogeneity problem. On the other hand, AMG estimator calculates both panel and cross-sectional coefficients and also takes the cross-sectional dependence unlike the first-generation estimators. Lastly, Dumitrescu and Hurlin (47) causality test considers the heterogeneity unlike the traditional Granger causality test and can produce relatively more robust findings under the presence of cross-sectional dependence.

## Results and Discussion

In the analysis part of the study, the pre-tests of cross-sectional dependence and heterogeneity among economic freedom, educational attainment and life expectancy are conducted. The cross-sectional dependence indicates that any shock in a country of the panel affects the other countries of the panel and cross-sectional dependence is widely seen in the highly integrated world (56). In this context, cross-sectional dependence is examined by using tests of *LM*_*adj*._, LM CD, and LM developed by Pesaran et al. (57–59), and the results of those tests are depicted in Table 3. The results of the cross-section dependence tests point out the cross-sectional dependence due to a decline in the null hypothesis at 1% as a consequence of the three tests in Table 3. For this reason, a unit root test, cointegration and causality tests, which give robust results under the entity of cross-sectional dependence, should be used. The homogeneity test checks whether the slope coefficient in the cointegration equation varies among the cross-sections. Therefore, the specification of homogeneity is important when selecting the causality and cointegration tests and estimator. The availability of homogeneity is explored by the homogeneity tests of Pesaran and Yamagata (60) and the findings of both tests are presented in Table 3. The null hypothesis (the entity of homogeneity) is rejected and the entity of heterogeneity is achieved.

**Table 3:** Results of cross-sectional dependence and heterogeneity tests.

**Test**	**Test statistic**	***P-*value**
LM_adj_	29.347	0.015
LM CD	30.991	0.009
LM	34.265	0.000
Δ^~^	19.453	0.003
$\Delta_{\text{\_}\text{adj}.}^{\sim }$	22.705	0.011

The stationarity of the series, in other words the presence of unit root in the series, can lead spurious regression and in turn decrease the reliability of the findings (61). Therefore, the availability of unit root in LNLIE, LNEF and LNEDU is investigated by using the Cross-Sectionally augmented (62) (CIPS) test proposed by Pesaran (63) due to cross-sectional dependence among the three series. The results of the test are presented in Table 4 and test statistics are compared with the critical values in Pesaran (63). Thus, the null hypothesis indicating the presence of a unit root in the series is accepted for level values of the series, because test statistics are found to be lower than the critical values. However, the null hypothesis (presence of a unit root in the series) is denied for the first differences of the variables, because test statistics are found to be higher than the critical values. In conclusion, test findings indicate that LNLIE, LNEF and LNEDU are I (1).

**Table 4:** Results of the CIPS unit root test.

**Variables**	**Level**		**First differences**	
	**Constant**	**Constant + Trend**	**Constant**	**Constant + Trend**
LNLEI	−1.342	−1.387	−8.335^**^	−9.012^**^
LNEF	−1.105	−1.329	−6.667^**^	−7.375^**^
LNEDU	−1.411	−1.503	−7.265^**^	−8.316^**^

** *It is significant at 5% significance level*.

The long-run interaction between economic freedom, educational attainment, and life expectancy is investigated by using the Westerlund and Edgerton (46) cointegration test due to the presence of heterogeneity and cross-sectional dependency. The cointegration test results are shown in Table 5. A significant cointegration relationship between economic freedom, educational attainment and life expectancy is obtained, because null hypothesis of significant cointegration relationship among three variables is accepted considering bootstrap *p*-values.

**Table 5:** Westerlund and Edgerton (46) bootstrap cointegration test.

**Constant**			**Constant** **+** **Trend**		
**Test statistic**	**Asymptotic *p*-value**	**Bootstrap *p*-value**	**Test statistic**	**Asymptotic *p*-value**	**Bootstrap *p*-value**
6.385	0.293	0.311	8.210	0.327	0.396

*Bootstrap critical values were generated from 10,000 repetitions, and asymptotic probability values were procured from standard normal distribution*.

The cointegration coefficients are calculated by using the AMG estimator, CCE-MG estimator and IFE estimator owing to the presence of cross-sectional dependency, heterogeneity, and robustness. The long run coefficients are presented in Table 6 and similar findings from three estimators verified the robustness of the AMG estimator, but the magnitude of the impact of economic freedom and education on life expectancy varies depending on the estimators. Both panel and country-level cointegration coefficients by three estimators indicate that economic freedom and educational attainment fostered life expectancy in the long run. The effect of educational attainment on life expectancy at the panel level and country-level is relatively higher when compared to economic freedom. On the other hand, the long-term effect of economic freedom and educational attainment on life expectancy varies among the countries. The positive effect of economic freedom on life expectancy is relatively higher in Hungary, Poland, and Bulgaria, but relatively lower in Czechia, Estonia, and Slovakia. This can be resulted from that Bulgaria made the largest improvement in economic freedom during the study period. Hungary and Poland experienced a similar improvement in economic freedom when compared with the other countries, but relatively larger impact of economic freedom on life expectancy was seen in these two countries. This can be resulted from that the channels which economic freedom affects the life expectancy are more effective in Hungary and Poland. Furthermore, the positive effect of educational attainment on life expectancy is relatively higher in Poland, Hungary, and Lithuania, but relatively lower in Croatia, Czechia, and Slovakia.

**Table 6:** Results of cointegration coefficients estimation.

**Countries**	**LNEF**			**LNEDU**		
	**AMG**	**CCE-MG**	**IFE**	**AMG**	**CCE-MG**	**IFE**
Bulgaria	0.183**	0.159*	0.131*	0.327*	0.302*	0.296*
Croatia	0.148**	0.138*	0.126**	0.214**	0.197*	0.183**
Czechia	0.125**	0.118**	0.112*	0.247*	0.215**	0.198*
Estonia	0.130**	0.124**	0.108**	0.319**	0.280**	0.284**
Hungary	0.211*	0.196*	0.182*	0.401**	0.345*	0.371*
Latvia	0.173**	0.163*	0.142**	0.297*	0.231*	0.193*
Lithuania	0.168**	0.134*	0.115*	0.330**	0.298*	0.270*
Poland	0.192*	0.177*	0.147*	0.417**	0.366**	0.368**
Romania	0.162**	0.141**	0.155**	0.348*	0.322*	0.280*
Slovakia	0.140**	0.120**	0.102*	0.250**	0.228**	0.203**
Slovenia	0.154*	0.134*	0.110*	0.266**	0.213**	0.208**
Panel	0.178**	0.156**	0.125**	0.325**	0.308**	0.256**

**, *It is respectively significant at 1 and 5% significance level.

The economic freedom is expected to influence the life expectancy through economic growth and development based on country specific characteristics, economic freedom can foster the life expectancy by procuring higher levels of healthcare, medical care, better access to nourishment and clean water, environmental quality, and raising the educational awareness of the individuals if the improvements in economic growth and development are accompanied by economic freedom (27). Otherwise, economic growth with weak institutional quality can negatively influence the life expectancy through higher income and education inequality and lower environmental quality (19). As a result, the net influence of economic freedom on life expectancy can differ based on country specific characteristics. In the related literature, only a few researchers have analyzed the effect of economic freedom on life expectancy in panel datasets with different income levels of countries through the regression method and reached a positive influence of economic freedom on life expectancy (19, 29–32), although a negative impact of economic freedom on life expectancy is also possible theoretically. However, none of the researchers have analyzed the country level interaction between economic freedom and life expectancy considering the country specific characteristics. A positive influence of economic freedom on life expectancy in all countries is revealed in line with the empirical studies, but the impact of economic freedom on life expectancy changes in countries due to countries' institutional and educational quality.

Educational attainment has been accepted as a crucial factor in explaining the differences in life expectancy among countries, because education can influence the life expectancy through many diverse channels such as economic growth and development, access to health-related information, employment of healthcare opportunities, planning horizon and risk perception at the subsequent period, raising the efficiency of health production (21–23, 25). The extensive literature about the influence of educational attainment proxied by different education indicators on life expectancy has mainly reached a positive relationship between two variables (5, 8, 26, 33–42). Furthermore, educational attainment together with real GDP per capita have been suggested as the dominant factors for explaining the differences in life expectancy among the countries (8, 21, 23). Our findings were found to be in accord with the related literature.

Lastly, the causality among economic freedom, educational attainment and life expectancy is analyzed by using the Dumitrescu and Hurlin causality test (47). The findings are presented in Table 7. The causality analysis reveals a one-way causal effect from educational attainment and economic freedom to life expectancy, but no significant causality from life expectancy to economic freedom and educational attainment is found. The findings indicate that both economic freedom and educational attainment are also significant determinants of life expectancy on the short run, but life expectancy has an insignificant effect on economic freedom and educational attainment.

**Table 7:** Results of the Dumitrescu and Hurlin (47) causality test.

**Null hypothesis**	**Test**	**Test statistics**	***P*-value**
D(LNEF) ↛ D(LNLIE)	Whnc	8.477	0.000
	Zhnc	9.113	0.000
	Ztild	9.585	0.000
D(LIE) ↛ D(LNEF)	Whnc	2.188	0.273
	Zhnc	2.476	0.314
	Ztild	3.103	0.410
D(LNEDU) ↛ D(LNLIE)	Whnc	6.473	0.000
	Zhnc	6.982	0.000
	Ztild	7.215	0.006
D(LIE) ↛ D(LNEDU)	Whnc	1.863	0.128
	Zhnc	1.945	0.130
	Ztild	2.110	0.138

The causality analysis unveiled that both factors have significant causes of life expectancy in the short term. In other words, the influence of improvements in economic freedom and educational attainment can have a significant influence on life expectancy in the short run. However, a comparative analysis for the findings of causality analysis considering the related literature cannot be made, because the empirical studies have mainly employed the regression approach.

## Conclusion

Life expectancy is a significant determinant of welfare and public health, the reason for which it represents a priority both for the national governments' policies and for the UN sustainable development goals. Life expectancy has considerably increased in the world, but significantly changed between countries. Therefore, identifying the factors that may influence the differences in life expectancy between countries will contribute to better national and international decisions of the policy-makers.

This article analyzed the effect of economic freedom together with educational attainment on life expectancy in the new EU member states, which passed through a significant economic and institutional transformation process, by using causality and cointegration tests. The study period was limited with 2000–2019 period thanks to the availability of yearly economic freedom data. The causality and cointegration analyses pointed out a significant effect of economic freedom and educational attainment on life expectancy in line with theoretical considerations and the related empirical literature. However, the effect of educational attainment on life expectancy was higher when compared to economic freedom. The higher effect of educational attainment can be explained through the fact that educational attainment fosters life expectancy through various direct and indirect channels such as innovation, technological development, entrepreneurship, human development, economic growth, and development.

The results of the study clearly indicate that market-oriented economic structures and educational attainment are significant determinants of life expectancy. Furthermore, higher economic growth with insufficient institutional and educational quality can negatively influence the life expectancy. Therefore, economic structures are important for life expectancy, a significant indicator of public health and development level of the countries. On the other hand, educational attainment is revealed to be more effective on life expectancy than economic freedom. Education can help individuals to be better informed and to make competent decisions related to many aspects of their lives, including decisions related to their own health care. There are direct and indirect paths through which education can influence life expectancy. It is well-known that better-educated individuals are more likely to adopt healthier lifestyles therefore incorporating healthy lifestyle education into the school curriculum can be a useful way to achieve behavior change. Also, higher levels of education will increase individuals' opportunities to be better paid and access better health care services and adopt a healthy lifestyle. The health sector alone cannot assure a good level of health for people, all sectors are interconnected therefore adopting and implementing coherent education policies and programmes can be crucial for the long-term development of this sector. For providing the best population health outcomes policymakers have to make decisions based on rigorous data and research evidence. Governments need to make efforts to find proper solutions to improve enrollment and mitigate dropout rates that usually have a negative impact on people's health conditions. Education increases knowledge and information, helps people to be more conscientious about their health status, about health habits that need to be adopted to maintain or improve health conditions, to adopt a positive attitude, generally speaking, to live a healthy lifestyle. Formal learning can be combined with informal learning to promote competencies necessary for each individual to improve their health.

The findings of the study expose how interlinked economic freedom, education and human health can be. Countries can improve their health by increasing educational attainment. Education can create circumstances for better health, on the other hand, poor health is more likely to put educational attainment at risk. Researchers and policy makers have mutual responsibilities for strengthening health. Our findings can increase awareness of the possibility that education can be associated with improved health. Future studies can be conducted to analyze the main or sub-components of economic freedom on life expectancy.

## Data Availability Statement

Publicly available datasets were analyzed in this study. This data can be found here: https://www.fraserinstitute.org/economic-freedom/map? tiongeozone = world&page = map&year = 2019 https://hdr.undp.org/en/content/human-development-index-hdi.

## Author Contributions

AM, GS, YB, and MG have made significant contribution to design of the article, analysis, writing, and discussion of the article. All authors approved the submitted article.

## Conflict of Interest

The authors declare that the research was conducted in the absence of any commercial or financial relationships that could be construed as a potential conflict of interest.

## Publisher's Note

All claims expressed in this article are solely those of the authors and do not necessarily represent those of their affiliated organizations, or those of the publisher, the editors and the reviewers. Any product that may be evaluated in this article, or claim that may be made by its manufacturer, is not guaranteed or endorsed by the publisher.
